# A New Look at “Filler Cells” in the Brain Reveals Their Role in Learning

**DOI:** 10.1371/journal.pbio.1001263

**Published:** 2012-02-14

**Authors:** Janelle Weaver

**Affiliations:** Freelance Science Writer, Glenwood Springs, Colorado, United States of America

## Abstract

In vivo and in vitro studies reveal that astrocytes, classically considered supportive cells for neurons, regulate synaptic plasticity in the mouse hippocampus and are directly involved in information storage.

**Figure pbio-1001263-g001:**
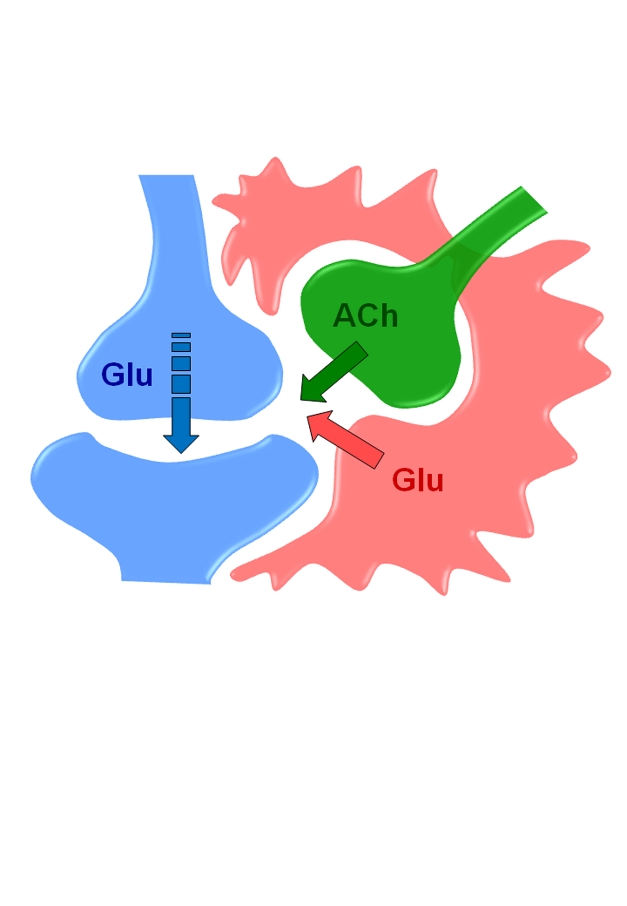
Astrocytes respond to active synapses and release transmitters that persistently enhance synaptic efficacy and thus play a direct role in synaptic plasticity and brain information storage.

[Fig pbio-1001263-g001]When we learn associations between items or ideas, such as the value of currency used in a foreign country, the connections between certain brain cells become stronger. In the cellular process thought to underlie learning and memory—long-term potentiation (LTP)—the simultaneous activation of two connected neurons causes one cell to respond more robustly to signals from the other one. “Neurons that fire together wire together” is a phrase commonly used to describe this phenomenon.

Recent studies suggest that neuronal communication and LTP are influenced by star-shaped glial cells called astrocytes, which are known to provide nutrients to neurons and support their basic functions. However, because much of this evidence was collected from brain slices and different research groups have produced conflicting results, scientists have questioned the involvement of astrocytes in learning.

This week in *PLoS Biology*, neuroscientist Alfonso Araque of the Cajal Institute in Madrid, Spain, and his collaborators report novel evidence of LTP regulation by astrocytes in living rats and propose a new cellular mechanism of learning and memory. Because this study suggests that astrocytes play an integral role in storing information in the brain, it resolves an important and high-profile debate in the field.

In the study, Araque and his team focused on pairs of neurons in the hippocampus, a brain region that is crucial for learning and memory. They generated LTP either by pinching the rat's tail or through direct electrical stimulation of the neurons.

Using in vivo imaging techniques, the researchers also found that these manipulations boosted calcium levels in astrocytes, causing them to release the neurotransmitter glutamate. This chemical signal binds to and activates proteins called metabotropic glutamate receptors (mGluRs) located on the surface of nearby neurons, thereby enhancing LTP. Consistent with this chain of events, the team found that elevated calcium levels in astrocytes triggered an increase in neurotransmitter release from the neurons.

The researchers further determined that LTP only occurred when mGluRs were functional and when neuronal responses and calcium signaling in astrocytes were simultaneously evoked by stimulation. By regulating LTP through the activation of mGluRs, which are prevalent in the hippocampus, astrocytes may support learning in a range of situations and could be an important target for treating Alzheimer's disease and other memory disorders.

**Navarrete M, Perea G, Fernandez de Sevilla D, Gómez-Gonzalo M, Núñez A, et al. (2012) Astrocytes Mediate In Vivo Cholinergic-Induced Synaptic Plasticity. doi:10.1371/journal.pbio.1001259**

